# Circulatory Level of Inflammatory Cytoskeleton Signaling Regime Proteins in Cancer Invasion and Metastasis

**DOI:** 10.3389/fonc.2022.851807

**Published:** 2022-07-07

**Authors:** Abhinay Kumar Singh, Atul Batra, Ashish Datt Upadhaya, Subhash Gupta, Haresh K P, Sharmistha Dey

**Affiliations:** ^1^ Department of Biophysics, All India Institute of Medical Sciences, New Delhi, India; ^2^ Medical Oncology, All India Institute of Medical Sciences, New Delhi, India; ^3^ Radiation Oncology, All India Institute of Medical Sciences, New Delhi, India; ^4^ Biostatistics, All India Institute of Medical Sciences, New Delhi, India

**Keywords:** breast cancer, biomarker, serum, metastasis, proteins

## Abstract

Early detection of metastatic breast cancer (MBC) is a serious issue for the healthcare system. It is essential to develop potential non-invasive, low-cost molecular biomarkers. The present study explored specific serum proteins of inflammatory, MAPK, and cytoskeletal signaling pathways involved in the progression of MBC to establish a panel of blood-based diagnostic and prognostic biomarkers. Healthy-control (HC), non-metastatic (NM), and metastatic (M) (pre- and post-therapy) breast cancer (BC) patients were recruited. LOX5, Rac1, Rac1b, p38α, phospho-p38α (Y182), LIMK1, phospho-LIMK1 (T508), cofilin1, and phospho-cofilin1 (S3) were quantified in the serum of the study group by real-time label-free surface plasmon resonance technology and verified by Western blot. Proteins were found to be significantly elevated in the serum of BC patients compared to HC and also higher in M compared to NM, which further downregulated in post-therapy M patients. Elevation of phospho-LIMK1 and phospho-cofilin1, which are critical for M, was also indicated in the serum level and can differentiate from NM. Receiver operating characteristics (ROC) derived area under the curve (AUC) (0.9) is very strong to differentiate between HC and BC. Moreover, the combined ROC of 3 molecules phospho-LIMK, p38α, and phospho-p38α were found to be a potent predictive panel of biomarkers between M and NM with AUC0.95. The panel of inflammatory cytoskeleton signaling regime proteins specified in this study can have significant clinical utility for diagnosis as well as prognosis of MBC at an early stage. The study may have a high translational value in a simple and cost-effective way by avoiding frequent CT/PET scans.

## Introduction

Breast cancer (BC) is the most common cancer in females across the globe and the second most prominent cancer in India (1, 2). Despite the development of various new drugs for the treatment of metastatic breast cancer, it remains an incurable disease. In the absence of any specific serum protein markers, imaging at frequent intervals is needed to monitor response to treatment and to confirm the suspicion of clinical progression. Identification of a serum protein marker for BC would potentially decrease the need for frequent CT and PET scans, thus decreasing the adverse effects of radiation and diagnostics costs.

Metastatic breast cancer (MBC) differs from the preceding primary BC in several properties. Primary cancerous tumor cells are surrounded by numerous cells like fibroblasts, macrophages, myeloid-derived suppressor cells, and neutrophils. These inflammatory cells produce pro-inflammatory factors and activate and induce epithelial to mesenchymal transition (EMT) and tumor metastasis by regulating the expression of transcription factors and proteases. Mesenchymal-like cells have properties that allow separation from the underlying connective tissue by diminishing intercellular contact by mesenchymal-like tumor and facilitating migration-like events, which causes metastasis (3). Cancer stem cells (CSC) play a key role in the formation of secondary tumors. CSC are resistant to therapeutic regimens. The origin of metastatic cells may be due to the transition of hematopoietic stem cells to CSC in tumor micro-environments (4). The inflammatory pathway - arachidonic acid cascade - promotes tumorigenesis and the lipoxygenase (LOX) enzyme directly regulates hematopoietic stem cell proliferation and function. Hence, due to the overproduction of LOX, hematopoietic cells transit to CSC (5).

LOX5 synthesizes hydroxyeicosatetraenoic acids (HETEs) and various leukotrienes (LTs), including leukotriene A4 (LTA4), leukotriene B4 (LTB4), leukotriene C4 (LTC4), leukotriene D4 (LTD4), and leukotriene E4 (LTE4) (6). LTB4, a 4 series of eicosanoids, is synthesized from unstable LTA4 by the action of LTA4 hydrolase. This is a powerful bioactive lipid that has been implicated in various physiological functions including inflammation, tumor promotion, and metastasis. The LTC4 is the product of LOX5 which binds with its receptor CysLT1, enhancing the expression of Tiam 1 (guanine exchange factor (GEF)) which further converts the inactive form of Rac1-GDP to the active form, Rac1-GTP. This active form phosphorylates and activates LIMKinase1 (LIMK1) through p38MAP Kinase. This active LIMK1 phosphorylate cofilin1, which no longer binds with F-actin and increases the polymerization of F-actin, causes metastasis. In normal physiological conditions, cofilin1 remains bound with F-actin and regulates its polymerization (7).

It is reported in our previous study and other studies that LOX5 protein expresses more in breast cancer (8–10). LOX5 was found to be over-expressed in non-metastatic BC patients and was downregulated after treatment. LOX5 and LOX 12 were found to be significantly higher in the patients with HER2+ breast cancer (11, 12). Leukotrienes produced by LOX5 activate the downstream molecules like Rac1, p38α MAPK, LIMK1, and cofilin1 which have a critical role in metastasis.

Since these molecules have a role in the development of metastatic cancer cells, the serum level may offer a new clinical approach to early diagnosis and monitoring of patients with MBC. The present assumption is that the panel of biomarkers would cumulatively diagnose the disease with higher sensitivity and specificity. The current study aims to evaluate the level of these proteins in metastatic disease and establish a panel of blood-based diagnostic and prognostic biomarkers.

## Materials and Methods

### Patients’ Recruitment

Study patients were recruited with clinically diagnosed 148 BC from the breast cancer clinic at Institute Rotary Cancer Hospital (IRCH), All India Institute of Medical Sciences (AIIMS), New Delhi, and 52 healthy controls (HC) were recruited by advertisement. Among the BC patients, 75 were categorized into non-metastatic (NM) and 73 metastatic (M) breast cancer patients. Ethics protocol (IECPG-191/27.03.2019) has been approved by the ethics subcommittee, AIIMS, New Delhi. Patients with any comorbidities such as hypertension, blood pressure, thyroid disease, severe infection, or who were already operated on for early BC were excluded from the study. Written consent was obtained from all the study participants.

Blood (5ml) was withdrawn from the study participants and HC. Serum was separated by centrifugation and stored at -80°C for further use.

The staging and TNM (Tumor, Node, and Metastasis) classification of disease was performed by the eight edition of the American Joint Committee on Cancer (AJCC) criteria. Demographic and clinical characteristics including age, menopause status, family history, habit (smoking and alcohol drinking), comorbidities, hormone receptor status, TNM status, and disease metastasis condition of study participants were addressed. Patients with metastasis were treated as per standard protocols by the treating medical oncologist and included hormonal therapy (aromatase inhibitor with/without a CDK4/6 inhibitor), taxane-based chemotherapy, taxane- and trastuzumab-based treatment for those with hormone-positive, triple-negative, and HER2 positive BC, respectively (13). Blood was collected twice: first, at the time of diagnosis from all study participants and second, after 3 months of treatment of metastatic patients only. Response to treatment was recorded by a CT or PET scan after 3 months and on the basis of the outcome of response, the patient was categorized in CR (complete response), PR (partial response), SD (stable disease), or PD (progressive disease) by the RECIST 1.1 criteria.

### Estimation of Inflammatory Cytoskeleton Signaling Protein in Serum of Study by SPR

Serum level of inflammatory cytoskeleton signaling proteins such as LOX5, Rac1, Rac1b, p38α, phospho-p38α (Y-182), LIMK1, phospho-LIMK1 (T-508), cofilin1, and phospho-cofilin1 (S-3) were assessed by using real-time label-free surface plasmon resonance (SPR) technology by Biacore 3000 instruments (GE Healthcare, Sweden). Antibody against all the above-mentioned proteins, i.e., Rabbit anti-human Rac1 IgG, Mouse anti-human p38α IgG, Mouse anti-human phospho-p38α (Y-182) IgG, (Santa Cruz Biotechnology, CA, USA), Rabbit anti-human LOX5 IgG (Cell Signaling Technology, Danvers, Massachusetts, USA), Rabbit anti-human Rac1b IgG (Merck Millipore, Burlington, Massachusetts, USA), Rabbit anti-human LIMK1 IgG, Rabbit anti-human phospho-LIMK1 (T-508) IgG, Rabbit anti-human cofilin1 IgG, and Rabbit anti-human phospho-cofilin (S-3) IgG (St. Louis, Immunotag USA) were immobilized on a separate flow cell of CM5 sensor chip by using amine coupling kit.

During the immobilization process, the dextran surface of the CM5 sensor chip was activated by applying a 1:1 v/v mixture of N-ethyl- N’-3diethylaminopropylcarbodiimide (EDC) (75 µg/µl) and N-hydroxysuccinimide (NHS) (12.5 µg/µl). After surface activation, antibodies in sodium acetate solution (pH 5.0, 10mM) were passed on to their respective flow cell for covalent immobilization and all the activated unreacted surface groups were blocked by the flow of ethanolamine (pH 8.0).

For generation of standard curve human recombinant LOX5, Rac1, Rac1b, p38α, LIMK1, and cofilin1 were cloned and expressed in bacterial expression system and purified by using Ni-NTA affinity column chromatography. Antigenic determinant of phospho-p38α (Y-182), phospho-LIMK1 (T-508), and phospho-cofilin1 (S-3) complementary to their respective antibody paratope were synthesized. The peptide sequence of antigenic determinant were procured from antibody datasheet and were synthesized by using Fmoc solid phase peptide (PS3) synthesis process (14). Standard curve was generated by flowing different known concentrations of purified proteins of LOX5 (0, 0.7, 1.75, 3.5, 7, 12.25, 17.5, 24.5, 35, 52.5, and 70 ng/μl), Rac1 (0, 0.135, 0.675, 1.35, 3.375, 4.725, 6.75, 10.125, and 13.5 ng/μl), Rac1b (0, 0.6, 1.2, 1.8, 2.4, 3.6, 4.8, 6, 9, and 12 ng/μl), p38α (0, 0.482, 0.96, 1.925, 2.89, 3.85, 9.625, 14.43, 19.25, 28.875, and 38.5 ng/μl), hosphor-p38α (Y-182) (0, 1.36, 3.42, 5.45, 6.81, 8.85, 10.9, 13.62, and 20.43 ng/μl), LIMK1 (0, 5.125, 10.25, 15.375, 20.5, 25.62, 38.4, 51.25, and 64.06 ng/μl), phospho-LIMK1 (T-508) (0, 2.3, 4.6, 6.9, 11.5, 13.8, 17.25, 23, 34.5, and 46 ng/μl), cofilin1 (0, 3.2, 5.6, 8, 12, 16, 24, 32, 40, 60, and 96 ng/μl), and phospho-cofilin1 (S-3) (0, 1.08, 2.175, 3.265, 4.35, 5.44, 8.15, 10.875, 16.31, and 21.75 ng/μl) on their respective antibody immobilized flow cell and Response Unit (RU) were obtained.

Correspondingly, serum samples of all study participants were passed over the antibody immobilized flow cell of CM5 sensor chip in dilution of 1:80 with HBS-EP buffer and RU were obtained. All these RU were plotted on respective standard curve and concentration of LOX5, Rac1, Rac1b, p38α, phospho-p38α (Y-182), LIMK1, phospho-LIMK1 (T-508), cofilin1, and phospho-cofilin1 (S-3) were calculated.

By execution of ROC analysis, cut-off value of LOX5, Rac1, Rac1b, p38α, phospho-p38α (Y-182), LIMK1, phospho-LIMK1 (T-508), cofilin1, and phospho-cofilin1 (S-3) proteins were calculated, which can be utilized as biomarkers to differentiate metastatic versus non-metastatic disease and healthy versus BC (NM+M) patients with high sensitivity and specificity of proteins related to the disease. We have also analyzed ROC curves with the panel of three sets of above proteins between NM and M. Three curves plotted are the combination of phospho-LIMK1, phospho-p38α and p38α (m1), LOX5, Rac1, phospho-p38α (m2) and LOX5, phospho-p38α, and hosphor-cofilin1(m3).

### Validation of Serum Level by Western Blot

A Western blot experiment was performed for validation of serum levels of all inflammatory cytoskeleton signaling proteins in all the study groups. A total of four serum samples were randomly selected from each group, i.e., HC, NM, M (pre-therapy and post-therapy group (MF)) for Western blot experiment. Serum albumin was removed by using albumin out kit (G-Biosciences, USA) and the total protein concentrations of albumin out of serum were determined by bicinchoninic acid assay (BCA) method. Protein samples were resolved by SDS-PAGE mini gel system and transferred to PVDF membrane (MDI, USA) by using standard protocol. Nonspecific sites were blocked by incubation of membrane in 5% non-fat dry milk solution in TBS-1 (10 mM Tris-Cl, 150 mM NaCl in miliQ water) for 2 hours. Blots were incubated with their primary antibodies and respective HRP conjugated secondary antibodies. Protein bands were detected by developing membranes in enhanced chemiluminescent system (Pierce ECL Western Blotting Substrate, Thermo Scientific, Rockford, IL, USA) and the band density was analyzed by myImageAnalysis™ software (Thermo Scientific).

### Statistical Analysis

Statistical analysis was performed by SPSS Statistics version 17.0 and Stata/IC version 11.1 (Stata Corp LP, College Station, TX, USA). Descriptive analysis of all variables was carried out and percentage, mean (95% confidence interval), and standard deviation (SD) were calculated as appropriate. Baseline comparisons between breast cancer patient of NM and M group and HC were made by using Student’s t-test for continuous variables, chi-square test for categorical variables, and ANOVA for comparison of more than two categories. For the determination of the best cut-off value for each protein, a receiver operating characteristic (ROC) curve was generated. Statistical significance was predefined at a level of p-value <0.05.

## Results

### Demographic and Clinicopathological Data

Demographic and clinical characteristics of 148 BC patients (75 NM and 73 M) and 52 HC are illustrated in **Table 1**. Out of 73 M patients, 44 underwent therapy, 19 died, and 10 discontinued their treatment. All study participants were female. The mean age of HC, NM, and M groups were 35.76, 48.49, and 45.83 years, respectively. Family history of different cancers like breast, ovarian, pancreatic, or prostate was present in 26.03% of the M group and 18.67% of the NM group of patients. In M and NM groups, 5.47% and 6.66% of patients, respectively, had habits of tobacco chewing, smoking, or alcohol drinking. According to TNM classification, the majority of M (49.31%) and NM (34.67%) group patients were recognized as T4 tumor size, and 35.61% and 32.00% were T3 in M and NM, respectively. Node involvement was recorded in 67.12% of M and 73.33% of NM group of patients. In the NM group, the majority were in stage III (66.66%), followed by stage II (33.34%). With respect to the hormonal profile, in the M group the majority were ER+ (61.64%) and PR+ (52.05%), and in the NM group of patients, 56.00% were ER+ and 45.33% were PR+. In the M group, HER2+ was found to be in 53.42% and in the NM group it was 48.00%.

**Table 1:** Demographic data of study groups.

Characteristics	Healthy Control n = 52	Non-metastatic n = 75	Metastatic n = 73
Gender			
Female	52 (100%)	75 (100%)	73 (100%)
Age (mean) y	35.76 ± 10.48	48.09 ± 10.44	45.83 ± 8.99
<30	12 (23.07%)	–	–
31-50	34 (65.38%)	44 (58.67%)	58 (79.45%)
51-70	6 (11.54%)	31 (41.33%)	15 (20.55%)
Menopause Status			
Pre	45 (86.54%)	37 (49.33%)	48 (65.75%)
Post	7 (13.46%)	38 (50.67%)	25 (34.25%)
Habit			
Non smoker		70 (93.33%)	69 (94.52%)
Smoker		5 (6.66%)	4 (5.47%)
Family History			
Absent	48 (92.31%)	61 (81.33%)	54 (73.97%)
Present	4 (7.69%)	14 (18.67%)	19 (26.03%)
Tumor Size	–		
T1		2 (2.66%)	–
T2		23 (30.66%)	11 (15.06%)
T3		24 (32.00%)	26 (35.61%)
T4		26 (34.67%)	36 (49.31%)
Node Status	–		
N0		20 (26.67%)	24 (32.88%)
N1		55 (73.33%)	49 (67.12%)
Stage	–		
II		25 (33.34%)	–
III		50 (66.66%)	–
IV		–	73 (100.00%)
ER status	–		
Positive		42 (56.00%)	45 (61.64%)
Negative		33 (44.00%)	28 (38.36%)
PR status	–		
Positive		34 (45.33%)	38 (52.05%)
Negative		41 (54.67%)	35 (47.95%)
HER2 status	–		
Positive		36 (48.00%)	39 (53.42%)
Negative		39 (52.00%)	34 (46.57%)

### Immobilization of LOX5, Rac1, Rac1b, p38α, Phospho-p38α (Y-182), LIMK1, Phospho-LIMK1 (T-508), Cofilin1, and Phospho-Cofilin1 (S-3) Antibody:

Antibodies of LOX5, Rac1, Rac1b, p38α, phospho-p38α (Y-182), LIMK1, phospho-LIMK1 (T-508), cofilin1, and phospho-cofilin1 (S-3) were successfully immobilized on flow cell of CM5 sensor chip and for each immobilization process RU of 1689, 2495.5, 4808.9, 3866.6, 1824.9, 4262, 3862.3, 1131.5, and 1297.4 were recorded, respectively, where 1 RU corresponds to 1pg/mm^2^ (**Figure S1**). Standard curves were generated for each protein by passing different concentrations of purified recombinant form on their respective flow cell and a linear graph was generated for every protein (**Figure S2**).

### Quantitative Estimation of LOX5, Rac1, Rac1b, p38α, Phospho-p38α (Y-182), LIMK1, Phospho-LIMK1 (T-508), Cofilin1, and Phospho-Cofilin1 (S-3) Protein in Serum of Study Groups by SPR

**Figure 1:**
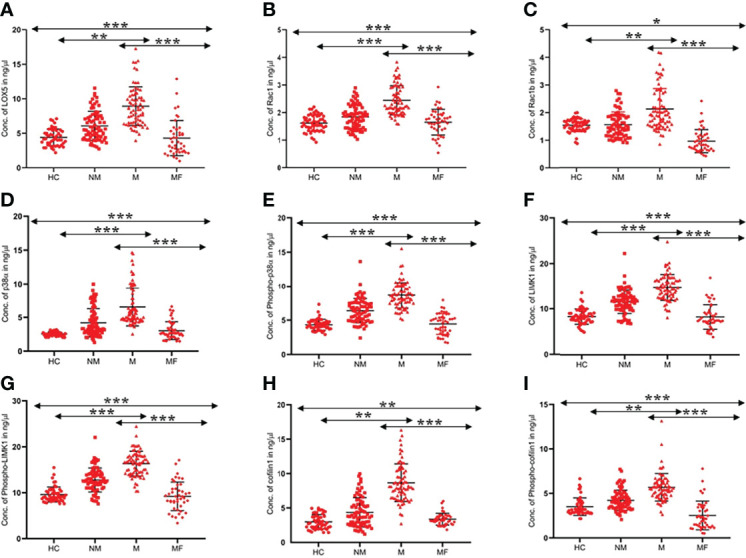
Scatter graph illustrated level of proteins **(A)**. LOX5, **(B)**. Rac1, **(C)**. Rac1b, **(D)**. p38a, **(E)**. Phospho-p38a **(F)** LIMK1 **(G)** Phospho-LIMK1 **(H)** Cofilin1 **(I)** Phospho-Cofilin1 in the serum of HC, NM, M, and MF (MF-metastatic post-therapy follow-up). For each group, standard error of the mean (mean ± SE) was plotted. ANOVA test was used to compare the means between the groups *- *p*<0.01, **- *p*<0.001, ***- *p*<0.0001.

The serum level of the above-mentioned proteins was found to be significantly (p<0.0001) higher in the M (N=73) group than in the NM (N=75) group and HC (N=52) group, as shown in **Figure 1**. The concentration of LOX5, Rac1, Rac1b, p38α, phospho-p38α (Y-182), LIMK1, phospho-LIMK1 (T-508), cofilin1, and phospho-cofilin1 (S-3) proteins in the M group were 9.11 ± 4.26 (95% CI: 8.12-10.11), 2.45 ± 0.64 (95% CI: 2.30-2.60), 2.12 ± 0.93 (95% CI: 1.90-2.34), 6.51 ± 3.15 (95% CI: 5.78-7.25), 8.65 ± 1.88 (95% CI: 8.21-9.08), 14.85 ± 3.49 (95% CI: 14.04-15.67), 16.31 ± 3.44 (95% CI: 15.51-17.11), 5.69 ± 1.55 (95% CI: 5.02-6.36), and 8.67 ± 4.62 (95% CI: 7.60-9.75) ng/µl, respectively; in the NM group were 6.04 ± 2.96 (95% CI: 5.36-6.73), 1.85 ± 0.49 (95% CI: 1.73-1.96), 1.49 ± 0.71(95% CI: 1.33-1.66), 4.19 ± 2.47 (95% CI: 3.62-4.76), 6.38 ± 1.84 (95% CI: 5.95-6.80), 11.60 ± 2.57 (95% CI: 11.01-12.19), 12.76 ± 2.60 (95% CI: 12.16-13.36), 4.23 ± 1.14 (95%CI: 3.94-4.52), and 4.38 ± 3.16 (95% CI: 3.65-5.11) ng/µl, respectively; and in the HC group were 4.39 ± 1.20 (95% CI: 4.05-4.73), 1.61 ± 0.29 (95% CI: 1.53-1.69), 1.59 ± 0.19 (95%CI: 1.53-1.64), 2.54 ± 0.31 (95%CI: 2.45-2.62), 4.31 ± 0.80 (95% CI: 4.09-4.54), 8.36 ± 1.68 (95% CI: 7.89-8.83), 9.59 ± 1.64 (95%CI: 9.13-10.05), 3.51 ± 0.99 (95% CI: 3.08-3.94), and 3.01 ± 1.80 (95% CI: 2.17-3.84) ng/µl, respectively (**Table 2**).

**Table 2:** Concentration of proteins in Healthy Control (HC), Non-metastatic (NM), Metastatic (M), and CR+PR of M group (pre- and post-therapy).

Protein	HC (n=52)	NM (n=75)	M (n=73)	p-value	CR+PR (Pre therapy) (n=31)	CR+PR (Post Therapy) (n=31)	p-value
LOX5	4.39±1.20	6.04±2.96	9.11±4.26	0.0001	8.24±3.02	3.47±1.68	0.0001
Rac1	1.61±0.29	1.85±0.49	2.45±0.64	0.0001	2.21± 0.54	1.39±0.35	0.0001
Rac1b	1.59±0.19	1.49±0.71	2.12±0.93	0.011	1.99±0.89	0.81±0.26	0.0001
p38α	2.54±0.31	4.19±2.47	6.51±3.15	0.0001	6.08±3.40	1.99±1.52	0.0001
Phospho-p38α	4.31±0.80	6.38±1.84	8.65±1.88	0.0001	8.61±1.96	2.40±0.86	0.0001
LIMK1	8.36±1.68	11.60±2.57	14.85±3.49	0.0001	14.55±2.95	5.28±1.53	0.0001
Phospho-LIMK1	9.59±1.64	12.76±2.61	16.31±3.44	0.0001	15.79±2.92	5.74±1.63	0.0001
cofilin1	3.51±0.99	4.23±1.14	5.69±1.55	0.001	5.57±1.34	2.80±0.52	0.0001
Phospho-cofilin1	3.01± 3.00	4.38±3.16	8.67±4.62	0.0001	8.16±4.05	1.18±0.63	0.0001

In the NM and M groups, the concentration of proteins increased gradually with tumor size and nodal involvement of disease. With respect to hormone status, protein levels were higher in the ER+, PR+, and HER2+ patients, as compared to their respective counterparts. No significant differences were observed in the concentration of proteins with age group, family history, or menopause status (**Table S1**).

### Serum Level of Proteins in Metastatic Group After Therapy

Blood was withdrawn after 3 months of treatment from the follow-up of the 44 metastatic patients; those who continued their treatment. The clinical and experimental evaluations were performed.

The concentration of LOX5, Rac1, Rac1b, p38α, phospho-p38α (Y-182), LIMK1, phospho-LIMK1 (T-508), cofilin1, and phospho-cofilin1 (S-3) proteins in pre-therapy condition were 8.22 ± 2.76, 2.23 ± 0.52, 1.93 ± 0.78, 5.89 ± 2.94, 8.42 ± 1.72, 14.27 ± 2.78, 15.53 ± 2.76, 5.47 ± 1.25, and 8.05 ± 3.56 ng/µl, respectively, and the concentration of proteins after therapy were decreased significantly to 4.29 ± 2.55, 1.64 ± 0.43, 0.96 ± 0.41, 3.01 ± .89, 4.43 ± 1.35, 8.34 ± 1.94, 9.35 ± 1.84, 3.35 ± 0.69, and 2.52 ± 1.64 ng/µl, respectively, with a p-value of 0.0001. The clinical assessment of these 44 patients (MF) reported that 31 completely/partially responded to the therapy and in the case of the rest of the 13 patients, the disease was stable/progressive. From our study with these 44 patients, the level of proteins decreased more significantly in patients with CR+PR as compared to the SD+PD group. The effect of therapy with other attributes was recorded and significant changes were observed (**Table S2**).

### ROC Curve

**Figure 2:**
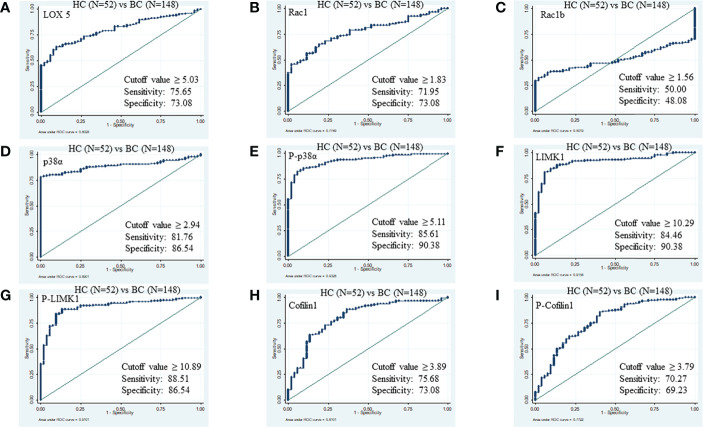
ROC of serum proteins between HC and BC. **(A)** LOX5; AUC: 0.802, **(B)** Rac1; AUC: 0.774, **(C)** Rac1b; AUC: 0.501, **(D)** p38α; AUC: 0.890, **(E)** phospho-p38α (Y-182); AUC: 0.932, **(F)** LIMK1, AUC: 0.915 **(G)** phospho-LIMK1 (T-508); AUC: 0.910, **(H)** Cofilin1; AUC: 0.810, **(I)** phospho-Cofilin1 (S-3); AUC: 0.772.

The ROC curve between HC (N=52) and BC (N=148) disease patients was generated for all proteins. The threshold cut-off value ≥5.03 ng/µl of LOX5 can distinguish BC disease from HC with area under the curve (AUC), sensitivity, specificity, positive predictive value (PPV), and negative predictive value (NPV) of 0.81, 75.65%, 73.08%, 88.7%, and 50.0%, respectively. Similarly, the cut-off values of Rac1, Rac1b, p38α, phospho-p38α (Y-182), LIMK1, phospho-LIMK1 (T-508), cofilin1, and phospho-cofilin1 (S-3) for BC disease were ≥1.83 (AUC 0.77, sensitivity 71.95%, specificity 73.08%, PPV 89.2%, NPV 48.8%), ≥1.561 (AUC 0.50, sensitivity 50.00%, specificity 48.08%, PPV 73.3%, NPV 25.3%), ≥2.94 (AUC 0.89, sensitivity 81.76%, specificity 86.54%, PPV 93.8%, NPV 62.0%), ≥5.11 (AUC 0.93, sensitivity 85.61%, specificity 90.38%, PPV 97.7%, NPV 72.1%), ≥10.29 (AUC 0.91, sensitivity 84.46%, specificity 90.38%, PPV 94.1%, NPV 67.7%), ≥10.89 (AUC 0.91, sensitivity 88.51%, specificity 86.54%, PPV 94.9%, NPV 72.6%), ≥3.89 (AUC 0.81, sensitivity 75.68%, specificity 73.08%, PPV 87.6%, NPV 50.0%), and ≥3.79 (AUC 0.77, sensitivity 70.27%, specificity 69.23%, PPV 87.5%, NPV 50.0%), respectively (**Figure 2**).

**Figure 3:**
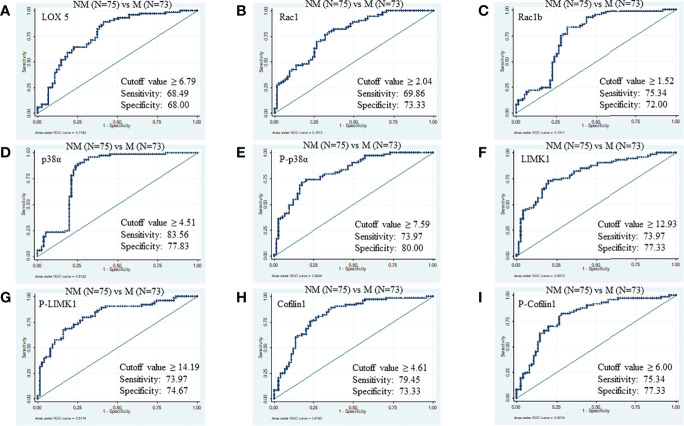
ROC of serum proteins between NM and M. **(A)** LOX5; AUC: 0.774, **(B)** Rac1; AUC: 0.781, **(C)** Rac1b; AUC: 0.757, **(D)** p38α; AUC: 0.812, **(E)** phospho-p38α (Y-182); AUC: 0.820, **(F)** LIMK1, AUC: 0.807 **(G)** phospho-LIMK1 (T-508); AUC: 0.817, **(H)** Cofilin1; AUC: 0.819, **(I)** phospho-Cofilin1 (S-3); AUC: 0.807.

Likewise, the cut-off values of LOX5, Rac1, Rac1b, p38α, phospho-p38α (Y-182), LIMK1, phospho-LIMK1 (T-508), cofilin1, and phospho-cofilin1 (S-3) to distinguish between M (N=73) from NM (N=75) were ≥6.79 (AUC 0.77, specificity 68.49%, sensitivity 68.00%, PPV 70.6%, NPV 79.4%), ≥2.04 (AUC 0.78, specificity 69.86%, sensitivity 73.33%, PPV 77.6%, NPV 80.6%), ≥1.52 (AUC 0.75, specificity 75.34%, sensitivity 72.00%, PPV 74.3%, NPV 75.7%), ≥4.51 (AUC 0.8, specificity 83.56%, sensitivity 77.83%, PPV 89.6%, NPV 94.4%), ≥7.59 (AUC 0.82, specificity 73.97%, sensitivity 80.00%, PPV 82.4%, NPV 83.8%), ≥12.93 (AUC 0.80, specificity 73.97%, sensitivity 77.33%, PPV 78.7%, NPV 80.8%), ≥14.19 (AUC 0.81, specificity 73.97%, sensitivity 74.67%, PPV 75.6%, NPV 80%), ≥4.61 (AUC 0.80, specificity 79.45%, sensitivity 73.33%, PPV 74.7%, NPV 79.7%), and ≥6.0 (AUC 0.81, specificity 75.3%, sensitivity 77.33%, PPV 82.9%, NPV 86.1%), respectively (**Figure 3**).

**Figure 4:**
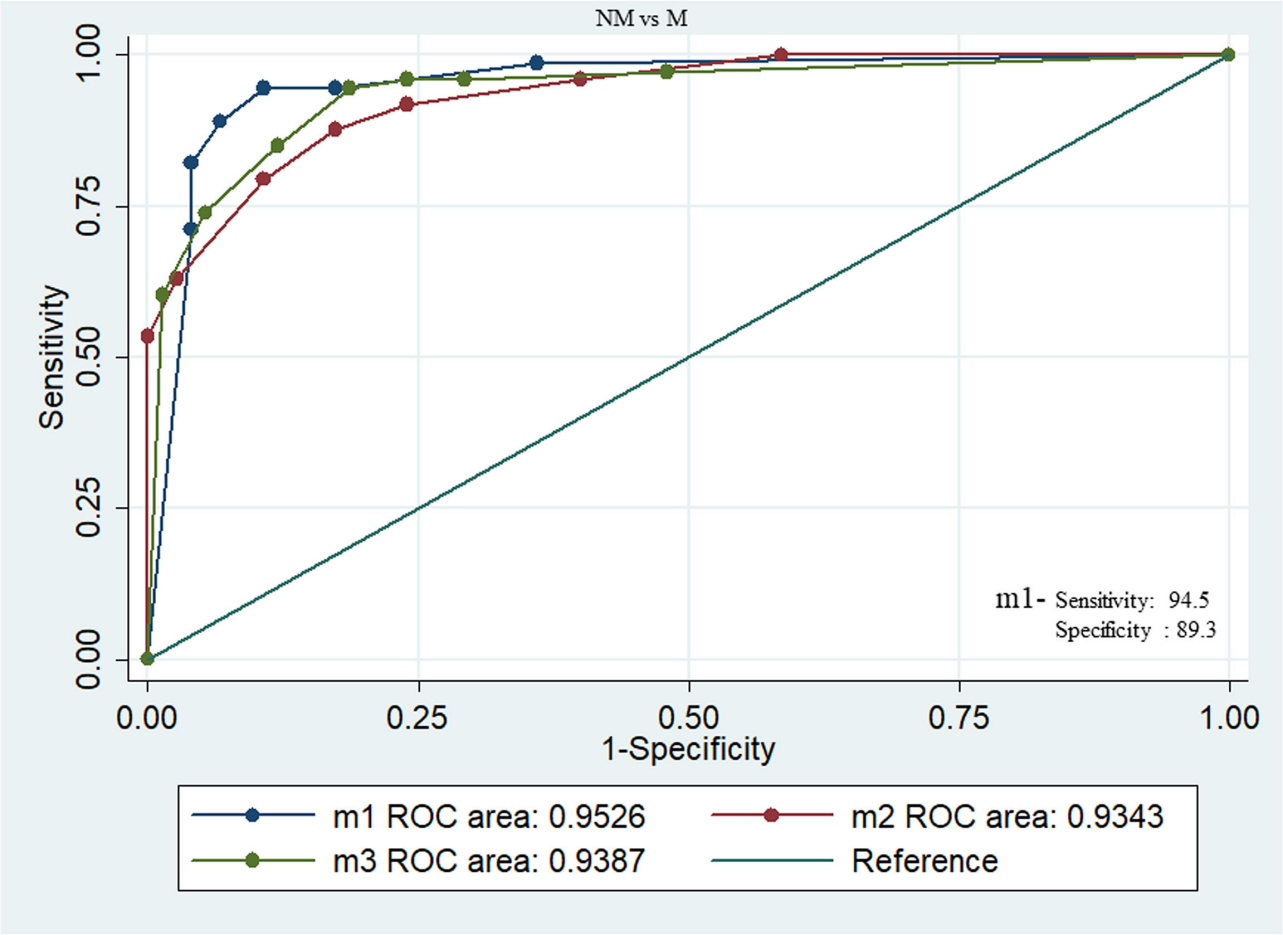
Combined ROC between NM and M breast cancer patients with panel of proteins m1(phospho-LIMK1, phospho-p38α, and p38α), m2 (LOX5, Rac1, and phospho-p38α), m3 (LOX5, phospho-p38α, and phospho-cofilin1). Among these combination m1 showing excellent AUC value of 0.95, specificity 94.5%, sensitivity 89.33%, PPV 89.6%, and NPV 94.4%.

We have also combined the ROC curves of three sets of above proteins to augment the best panel of protein marker for diagnosis between NM (N=75) and M (N=73). The combination of phospho-LIMK1, phospho-p38α, and p38α (m1) panels can produce a diagnostic marker for NM and M disease with an excellent AUC value of 0.95, specificity 94.5%, sensitivity 89.33%, PPV 89.6%, and NPV 94.4%. Similarly, the m2 (LOX5, Rac1, phospho-p38α) panel and m3 (LOX5, phospho-p38α, phospho-cofilin1) panel can also discriminate between NM and M disease status with a high AUC value of 0.93 (**Figure 4**).

### Validation by Western Blot

**Figure 5:**
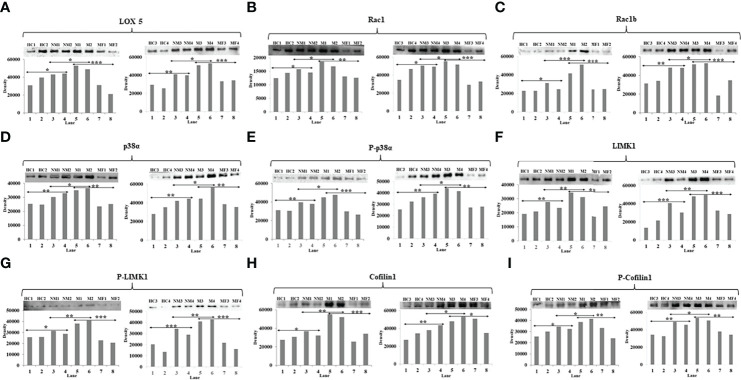
Western blot image and their densitometry analysis of proteins **(A)** LOX5, **(B)** Rac1, **(C)** Rac1b, **(D)** p38α, **(E)** phospho-p38α (Y-182), **(F)** LIMK1, **(G)** phospho-LIMK1 (T-508), **(H)** Cofilin1, **(I)** phospho-Cofilin1 (S-3) between HC, NM, M, and MF (Lanes-1&2-HC, 3&4-NM, 5&6-M, and 7&8-MF). An unpaired t-test was used to compare the means between two groups (**p<0.01 and *p<0.05).

Western blots were performed with four HC, four NM patients, four M patients, and four post-therapy (MF) metastatic patients to validate the expression level of LOX5, Rac1, Rac1b, p38α, phospho-p38α (Y-182), LIMK1, phospho-LIMK1 (T-508), cofilin1, and phospho-cofilin1 (S-3) proteins. The band density was seen in a similar pattern of SPR outcomes. Band densities of the HC group was significantly lower compared to BC patients. The band density of M patients was 2- to 3-fold higher in case of M compared to NM. The intensity of band was found to be 3-fold decreased in most of the cases in post-therapy M patients compared to pre-therapy patients (**Figure 5**).

## Discussion

MBC is the second leading cause of death in women worldwide (15). In patients with BC, the tumor cells may remain dormant in the secondary site for a decade and no clinical symptoms develop during this period (16). The clinical guidelines do not endorse any imaging or blood tests to be done routinely at follow-up of patients with early BC. Therefore, it becomes challenging to diagnose metastatic disease at an early stage due to the lack of suitable biomarkers. The present study explored the specific molecules in inflammatory, MAPK, and cytoskeletal signaling pathways involved by interlinkages with each stage of progression of MBC.

According to a WHO report, the incidence of BC and mortality is increasing rapidly. A total of 22,61,419 BC cases have been reported in 2020 in women worldwide (17), among which 2,05,424 cases were reported in India (18). One-third of these patients were diagnosed with MBC. Ninety percent of BC patients are hormone positive, and a majority have excess HER2+ protein. HER2+ protein increases the cell proliferation and raises the chances of developing metastasis (19). In our prospective case-control study, the percentage of ER+ and HER2+ are higher in M compared to NM.

In our study group, the M group patients are predominantly in the age group of 30-50 yrs, which supports other study findings (20, 21) suggesting that middle-age group women are more vulnerable to BC development.

In the inflammatory pathway, overproduction of LOX proteins increases hematopoietic cell proliferation and may transit to CSC which enter the circulatory system and develop metastasis (5). Our study observed the significant enhancement of LOX5 in the blood of M compared to NM patients.

Due to the over-production of LOX5, leukotriene (LTC4), the inflammatory cytokine mediator produced by LOX5 increases, which regulates epidermal growth factor (EGF). Further, it leads to an increase in the activation of Rac1 through Tiam1 expression that induces cancer cell migration toward metastasis (22). The same result is reflected at the circulatory level in our study - Rac1, and its mutated form Rac1b, both were found to be present in increased levels in the blood of M patients compared to NM.

The MAPKKK is typically activated by interactions with a small GTPase Rac1 and/or phosphorylation by protein kinases downstream from cell surface receptors. The activity of LIMK1 is also regulated mainly by GTPases Rac1 through their downstream kinases MAPKAPK2 (MK2), PAK1, and PAK4 (23, 24) that phosphorylate LIMK1&2 on a threonine residue T508 and T505, respectively (25). LIMkinases control microtubule dynamics, independent of their regulation of actin microfilament, and maintain the balance of polymerization and depolymerization of actin through cofilin protein. Cofilin activity is regulated through its phosphorylation on Ser3 mainly by LIMK1&2 (26–28).

On phosphorylation at Ser3, cofilin1 loses its ability to bind to and depolymerize F-actin, resulting in increased polymerized F-actin levels (29). In normal conditions, cofilin1 remains bound with F-actin and regulates its polymerization and depolymerization phenomena. An increase in LIMK1 phosphorylate cofilin1, which no longer binds with F-actin, upsurges the polymerization of F-actin, which causes lamellipodia, filopodia formation, and movement of cells toward metastasis. Due to this alteration, CTC moves to a different part of the body. This variation in molecular activity is linked to MAPK signaling pathways. Interestingly, these changes were indicated in our present work. We found the concentration level of p38α, phospho-p38α, LIMK1, phospho-LIMK1, and cofilin1 in the serum of M patients was significantly higher as compared to NM patients. Due to the overproduction of phospho-LIMK1 it phosphorylates cofilin1 at a very high level and phospho-cofilin1 was found in increased levels in the serum of M compared to NM patients.

In the present study, ROC analysis was done to develop these molecules as biomarkers to distinguish the BC patients from healthy controls at an early stage as well as from metastatic stage. Among the molecules of three types of interlinked pathways, p38α, phospho-p38α, LIMK1, phospho-LIMK1, and cofilin1 were found to be the best to diagnose BC with more than 0.9 area under the curve with high sensitivity and specificity. However, AUC derived from ROC between NM and M was 0.8 which is not as strong as shown between HC and BC.

Hence, a model of ROC by combining three different molecules in one set was generated and AUC was calculated to establish a panel of the best combination of molecules for the diagnostic biomarkers of M patients. The combination of the three molecules (m1) phospho-LIMK1, p38α, and phospho-p38α were found to be a very strong predictive panel of biomarkers between M and NM with AUC 0.95 and cut point 6.6.

There was not much difference observed in the expression level of all the experimental proteins with baseline characteristics such as age, menopause status, family history, and habit between the M and NM groups.

The M patients with ER+ were found to have significantly enhanced protein levels compared to ER- M patients. Similarly, over-expressed proteins were observed with HER2+ compared to HER2- in M patients.

It was reported that upregulation of HER2+, a member of the EGFR family (epidermal growth factor receptor) is directly associated to the aggressiveness of BC. Tyrosine phosphorylation and activation of the HER2+ receptor causes activation of ras/MAP kinase signal transduction pathways in breast cancer cells (30). ER+ and HER2+ BC patients were found with over-expressed p38 MAPK and tamoxifen resistance in an earlier study (31). Interestingly, in our study, it was observed that levels of Rac1 and p38αMAPK were higher in the serum of metastatic HER2+ BC. Hence, we can say LOX5 together with other cytoskeletal signaling molecules mentioned in this study have a role in the development of MBC in both hormonal groups. However, HER2+ BC patients with an elevated level of these proteins have a high risk of developing metastasis.

The protein levels were estimated after 3 months of therapy for 44 patients who continued the treatment and were found to be significantly downregulated. But after differentiating 44 post-therapy patients in term of clinical responder, 31 were found CR+PR and 13 were SD+PD. All these protein molecules in 31 CR+PR patients were found to be significantly downregulated. No significant downregulation was observed in the case of SD+PD patients. Surprisingly, the concentration of phospho-LIMK and phospho-cofilin1 in M patients decreased below the normal value after therapy, which indicates that these molecules provoke the cell motility for metastasis and can be a potent prognostic blood-based biomarker for M patients.

There are two clinical scenarios where the utility of these biomarkers, either as individual or a panel of multiple molecules, can be considered and another for screening the population for BC. Annual or biennial mammograms are the current standard for population-based screening of BC (32). However, these are uncomfortable and logistically challenging in the resource-limited settings (33), and a blood-based marker may increase the uptake of population for BC screening.

Second, patients with NM are followed up clinically after completion of treatment and further investigations are considered when clinically indicated. Therefore, a blood-based marker or panel may assist in detecting early metastatic disease in such patients in clinics.

This can be concluded that the panel of proteins specified in this study can serve as a novel serum protein marker for diagnosis of early M as well as the prognosis of M by real-time label-free method using surface plasmon resonance technology. This may help to monitor the disease in a simple way and can avoid frequent CT and PET scans. The study can be proposed to have high translational value to clinical practice.

## Data Availability Statement

The raw data supporting the conclusions of this article will be made available by the authors, without undue reservation.

## Ethics Statement

The studies involving human participants were reviewed and approved by All India Institute of Medical Sciences Ethics Committee (AIIMS) approved the study protocol (IECPG-191/27.03.2019). The patients/participants provided their written informed consent to participate in this study.

## Author Contributions

AS: performed all the experimental work, wrote methods & results. AB: diagnosed patients, supervision, verification of data and contributed for manuscript writing, editing. AU: Statistical analysis, HP & SG: recruited patients for radiotherapy, SD: Conceptualization, resources, supervision, verification of data, writing original manuscript, project administration, writing introduction, review, discussion and editing. All authors contributed to the article and approved the submitted version.

## Funding

This study is Ph.D. thesis work partially funded by the home institute of the consumable items

## Conflict of Interest

The authors declare that the research was conducted in the absence of any commercial or financial relationships that could be construed as a potential conflict of interest.

## Publisher’s Note

All claims expressed in this article are solely those of the authors and do not necessarily represent those of their affiliated organizations, or those of the publisher, the editors and the reviewers. Any product that may be evaluated in this article, or claim that may be made by its manufacturer, is not guaranteed or endorsed by the publisher.

## Supplementary Material

Supplementary Table 1
**(A, B)**. The concentration of proteins as mean and standard deviation with different attributes in NM and M patients.

Supplementary Table 2 The concentration of proteins in pre- and post-therapy metastatic patients with different attributes.

Supplementary Figure 1 Immobilization of LOX5, Rac1, Rac1b, p38α, hosphor-p38α (Y-182), LIMK1, hosphor-LIMK1 (T-508), cofilin1, and phospho-cofilin1 (S-3) antibodies on CM5 sensor chip.

Supplementary Figure 2 Standard curve of LOX5, Rac1, Rac1b, p38α, phospho-p38α (Y-182), LIMK1, phospho-LIMK1 (T-508), cofilin1, and phospho-cofilin1 (S-3) proteins.
