# Supplementary material for: Circulatory Level of Inflammatory Cytoskeleton Signaling Regime Proteins in Cancer Invasion and Metastasis
Source: Front Oncol. 2022 Jul 7;12:851807. doi: 10.3389/fonc.2022.851807 (PMC9300851; doi:10.3389/fonc.2022.851807)
Supplement: Supplementary Table 1 — (A, B). The concentration of proteins as mean and standard deviation with different attributes in NM and M patients. [file Table_1.docx]

| Characteristics | LOX5 | | | | Rac1 | | Rac1b | | p38α | | Phospho-p38α | |
| --- | --- | --- | --- | --- | --- | --- | --- | --- | --- | --- | --- | --- |
|  | NM | | M | | NM M | | NM M | | NM M | | NM M | |
| Age-  31-50  51-70  p-value | n=44  n=31 | 6.13±2.83  5.92±3.17  0.3838 | n=58  n=15 | 9.17±4.57  8.89±2.87  0.4094 | 1.91±0.55  1.76±0.40  0.0906 | 2.43±0.67  2.53±0.51  0.7061 | 1.44±0.68  1.57±0.75  0.7778 | 2.15±1.04  2.08±0.91  0.1603 | 3.94±2.43  4.53±2.53  0.8437 | 6.63±3.49  6.32±2.30  0.0251 | 6.36±1.94  6.40±1.71  0.5404 | 8.61±1.81  8.79±2.18  0.6333 |
| Menopause-  Pre  Post  p-value | n=37  n=38 | 6.22±2.92  5.87±3.03  0.3053 | n=48  n=25 | 9.57±4.88  8.23±2.57  0.1019 | 1.92±0.57  1.78±0.40  0.1019 | 2.40±0.59  2.54±0.73  0.8060 | 1.43±0.70  1.56±0.72  0.8624 | 2.20±1.02  1.97±0.74  0.1688 | 3.87±2.55  4.50±2.39  0.7746 | 6.66±3.29  6.22±2.89  0.2889 | 6.34±2.033  6.42±1.66  0.5755 | 8.57±1.94  8.80±1.78  0.6918 |
| Habit-  Non-smoker  Smoker  p-value | n=70  n=5 | 6.01±2.86  6.24±3.61  0.5921 | n=69  n=4 | 5.73±2.04  12.50±7.97  0.5881 | 1.83±0.511.94±.38  0.7359 | 1.90±0.57  3.33±0.62  0.0029 | 1.49±0.71  1.50±0.72  0.5182 | 2.58±0.94  2.33±1.24  0.8075 | 4.25±2.56  3.84±1.95  0.3073 | 6.65±3.26  6.37±2.43  0.8737 | 6.49±1.90  5.76±1.33  0.1157 | 7.94±1.73  9.35±4.27  0.8150 |
| Family History-  Absent  Present  p-value | n=61  n=14 | 6.30±3.14  4.94±1.63  0.0610 | n=54  n=19 | 9.32±4.46  8.53±3.68  0.2464 | 1.81±0.51  2.01±0.40  0.9129 | 2.43±0.65  2.51±0.62  0.6654 | 1.57±0.74  1.19±0.41  0.0359 | 2.17±0.98  1.97±0.81  0.2155 | 4.38±2.64  3.32±1.33  0.0746 | 6.62±3.28  6.21±2.81  0.3185 | 6.38±1.93  6.38±1.45  0.4969 | 8.78±1.88  8.26±1.86  0.1525 |
| Tumor Size-  T1+T2  T3  T4  p-value | n=25  n=24  n=26 | 4.61±1.31  5.74±2.14  7.76±3.88  0.000 | n=11  n=26  n=36 | 6.90±1.72  8.55±2.88  10.20±5.26  0.0001 | 1.82±0.40  1.83±0.64  1.90±0.40  0.023 | 2.27±0.51  2.48±0.56  2.49±0.73  0.242 | 1.19±0.48  1.39±0.52  1.89±0.87  0.005 | 1.64±0.31  1.95±0.77  2.39±1.09  0.0001 | 3.52±2.273.74±1.67  5.24±2.97  0.025 | 4.85±0.72  5.93±2.82  7.44±3.54  0.0001 | 5.62±1.61  6.32±1.627.16±1.97  0.511 | 7.93±1.65  8.99±1.62  9.06±1.99  0.053 |
| Node-  N0  N1  p-value | n=25  n=50 | 5.91±2.83  6.09±3.03  0.5929 | n=24  n=49 | 8.16±2.69  9.58±4.81 | 1.79±0.471.87±0.51  0.7086 | 2.26±0.44  2.55±0.70  0.9656 | 1.43±0.67  1.52±0.73  0.6673 | 1.88±0.69  2.24±1.02  0.9410 | 4.00±2.74  4.25±2.39  0.6489 | 5.65±2.14  6.93±3.48  0.9482 | 6.20±1.39  6.44±1.98  0.6906 | 8.01±1.23  8.96±2.06  0.9798 |
| Stage-  II  III  IV  p-value | n=25  n=50 | 5.56±2.36  6.29±3.21  0.8398 | n=73 | 9.11±4.260.0001 | 1.77±0.40  1.88±0.53  0.8136 | 2.45±0.64  0.0001 | 1.28±0.48  1.60±0.78  0.9671 | 2.12±0.93  0.0001 | 3.40±1.364.58±2.81  0.9735 | 6.51±3.15  0.0001 | 6.03±1.84  6.56±1.83    0.8781 | 8.65±1.88  0.0001 |
| ER Status-  E+  ER-  p-value | n=42  n=33 | 6.52±3.56  5.43±1.82  0.0576 | n=45  n=28 | 10.18±4.93  7.39±1.97  0.0029 | 1.89±0.531.80±0.45  0.2249 | 2.52±0.68  2.34±0.55  0.1261 | 1.60±0.81  1.36±0.54  0.0808 | 2.29±1.02  1.84±0.70  0.0231 | 4.40±2.60  3.92±2.31  0.2052 | 6.98±3.30  5.76±2.79  0.0551 | 6.52±2.0746.20±1.50  0.2241 | 8.93±1.94  8.18±1.70  0.0491 |
| PR Status-  PR+  PR-  p-value | n=34  n=41 | 6.42±3.32  5.73±2.62  0.1617 | n=38  n=35 | 10.23±4.95  8.08±3.24  0.0153 | 1.87±0.571.83±0.43  0.3424 | 2.56±0.70  2.35±0.56  0.0781 | 1.58±0.81  1.42±0.61  0.1630 | 2.29±1.04  1.97±0.80  0.0747 | 4.52±3.00  3.91±1.94  0.1476 | 6.84±3.37  6.21±2.95  0.1968 | 6.43±1.816.34±1.88  0.4117 | 8.81±1.82  8.51±1.94  0.2552 |
| HER2 Status-  HER2+  HER2-  p-value | n=36  n=39 | 6.02±2.69  6.06±3.22  0.5243 | n=39  n=34 | 10.21±5.33  7.86±2.49  0.0513 | 1.83±0.461.87±0.53  0.6350 | 2.64±0.74  2.26±0.52  0.0146 | 1.42±0.54  1.56±0.84  0.7966 | 2.32±1.16  1.89±0.63  0.0862 | 3.78±1.58  4.56±3.05  0.9134 | 7.2±3.82  5.78±2.33  0.0608 | 6.27±1.476.48±2.14  0.6929 | 9.43±1.98  7.82±1.76  0.0005 |

**Table S1 A: Concentration of proteins as mean and standard deviation with different attributes in NM and M patients**

**Table S1 B**: **Concentration of proteins as mean and standard deviation with different attributes in NM and M patients**

| Characteristics | LIMK | | | | Phospho-LIMK1 | | cofilin1 | | Phospho-cofilin1 | |
| --- | --- | --- | --- | --- | --- | --- | --- | --- | --- | --- |
|  | NM | | M | | NM M | | NM M | | NM M | |
| Age-  31-50  51-70  p-value | n=44  n=31 | 11.83±2.93  11.28±1.94  0.1821 | n=58  n=15 | 15.06±3.62  14.05±2.90  0.1608 | 13.04±2.93  12.35±2.02  0.1302 | 16.46±3.54  15.72±3.54  0.2317 | 4.46±1.24  3.90±0.91  0.0184 | 5.80±1.64  5.26±1.02  0.1139 | 5.16±3.54  3.27±2.12  0.0049 | 8.93±2.04  7.69±2.23  0.1794 |
| Menopause  Pre  Post  p-value | n=37  n=38 | 11.69±3.13  11.51±1.92  0.3834 | n=48  n=25 | 15.05±3.85  14.49±2.68  0.2598 | 13.01±3.11  12.53±2.01  0.2198 | 16.45±3.72  16.04±2.88  0.3163 | 4.51±1.32  3.96±0.88  0.0178 | 5.80±1.73  5.49±1.11  0.2160 | 5.34±3.74  3.44±2.14  0.0043 | 8.89±5.30  8.25±2.93  0.2892 |
| Habit-  Non-smoker  Smoker  p-value | n=70  n=5 | 11.68±2.65  11.15±2.12  0.2688 | n=69  n=4 | 14.04±3.57  15.67±2.97  0.5307 | 12.79±2.67  12.59±2.26  0.4112 | 14.63±3.51  18.01±2.93  0.5900 | 4.19±1.144.43±1.17  0.7299 | 4.85±1.51  6.54±2.67  0.7607 | 4.43±3.23  4.09±2.87  0.3751 | 6.15±3.41  11.20±5.67  0.6864 |
| Family History-  Absent  Present  p-value | n=61  n=14 | 11.62±2.71  11.52±1.91  0.4496 | n=54  n=19 | 14.93±3.81  14.65±2.48  0.3831 | 12.74±2.72  12.82±2.11  0.5400 | 16.39±3.69  16.08±2.68  0.3706 | 4.25±1.204.13±0.89  0.3556 | 5.77±1.68  5.46±1.07  0.2274 | 4.41±3.31  4.23±2.49  0.4234 | 8.98±5.03  7.82±3.12  0.1753 |
| Tumor Size-  T2  T3  T4  p-value | n=25  n=24  n=26 | 10.64±2.20  11.48±2.28  12.63±2.84  0.382 | n=11  n=26  n=36 | 13.73±2.41  14.42±2.42  15.80±4.16  0.008 | 11.85±2.41  12.74±2.26  13.64±2.85  0.498 | 15.34±2.74  15.51±2.31  17.26±3.95  0.051 | 3.66±0.89  4.48±1.04  4.54±1.28  0.2 | 5.09±0.92  5.45±1.22  6.05±1.82  0.017 | 2.84±2.36  5.14±3.14  5.15±3.40  0.196 | 6.92±2.85  7.80±3.76  9.84±5.34  0.034 |
| Node-  N0  N1  p-value | n=25  n=50 | 11.44±1.82  11.66±2.81  0.6246 | n=24  n=49 | 14.57±2.01  14.99±4.03  0.6856 | 12.56±1.97  12.83±2.81  0.6556 | 15.95±2.21  16.49±3.92  0.7342 | 4.30±1.014.20±1.19  0.3739 | 5.48±1.09  5.80±1.73  0.7929 | 4.23±2.28  4.43±3.44  0.5940 | 8.03±3.31  8.99±5.14  0.7973 |
| Stage-  II  III  IV  p-value | n=25  n=50 | 10.99±2.17  11.90±2.72  0.92 | n=73 | 14.85±3.49  0.0001 | 12.17±2.38  13.05±2.68  0.9146 | 16.31±3.44  0.0001 | 4.01±1.04  4.34±1.19  0.8735 | 5.69±1.55  0.001 | 3.95±2.77  4.59±3.34  0.7928 | 8.67±4.62  0.0001 |
| ER Status-  ER+  ER-  p-value | n=42  n=33 | 11.73±2.91  11.43±2.09  0.3091 | n=45  n=28 | 15.43±3.80  13.92±2.73  0.0361 | 12.84±2.88  12.66±2.23  0.3868 | 16.97±3.71  15.25±2.71  0.0186 | 4.27±1.21  4.18±1.07  0.3747 | 6.09±1.75  5.05±0.84  0.0022 | 4.37±3.34  4.39±2.97  0.5069 | 9.93±5.21  6.66±2.43  0.0013 |
| PR Status-  PR+  PR-  p-value | n=34  n=41 | 11.52±2.76  11.67±2.41  0.6005 | n=38  n=35 | 15.53±4.20  14.23±2.57  0.0561 | 12.69±2.78  12.81±2.50  0.5752 | 16.98±4.02  15.71±2.71  0.0562 | 4.22±1.23  4.24±1.08  0.5274 | 5.90±1.93  5.51±1.07  0.1347 | 4.21±3.42  4.52±2.96  0.6599 | 9.34±5.79  8.06±3.14  0.1190 |
| HER2 Status-  HER2+  HER2-  p-value | n=36  n=39 | 11.50±1.95  11.69±3.06  0.6204 | n=39  n=34 | 15.85±3.97  13.82±3.00  0.0163 | 12.70±2.06  12.81±3.05  0.5743 | 17.34±3.72  15.23±3.11  0.0111 | 4.07±0.97  4.38±1.27  0.8783 | 6.04±1.83  5.32±1.23  0.0708 | 4.07±2.60  4.66±3.62  0.7910 | 9.85±5.67  7.46±3.31  0.0344 |

**Table S2. Concentration of proteins in pre and post therapy metastatic patient with different attributes.**

| **Characteristics** | **LOX5** | **Rac1** | **Rac1b** | **p-38α** | **Phospho-p38α** | **LIMK1** | **Phospho-LIMK1** | **cofilin1** | **Phospho-cofilin1** |
| --- | --- | --- | --- | --- | --- | --- | --- | --- | --- |
| Pre therapy (n=44)  Post therapy (n=44)  p-value | 8.22±2.76  4.29±2.55  0.0001 | 2.23± 0.52  1.64± 0.43  0.0001 | 1.93±0.78  0.96± 0.41  0.0001 | 5.89±2.94  3.01±0.89  0.0001 | 8.42±1.72  4.43±1.35  0.0001 | 14.27±2.78  8.34±1.94  0.0001 | 15.53±2.76  9.35±1.84  0.0001 | 5.47±1.25  3.35±0.69  0.0001 | 8.05±3.56  2.52±1.64  0.0001 |
| CR+PR (n=31)  Pre therapy  Post therapy  p-value | 8.24±3.02  3.47±1.68  0.0001 | 2.21± 0.54  1.39±0.35  0.0001 | 1.99±0.89  0.81±.26  0.0001 | 6.08±3.40  1.99±1.52  0.0001 | 8.61±1.96  2.40±0.86  0.0001 | 14.55±2.95  5.28±1.53  0.0001 | 15.79±2.92  5.74±1.63  0.0001 | 5.57±1.34  2.8±0.52  0.0001 | 8.16±4.05  1.18±0.63  0.0001 |
| SD+PD (n=13)  Pre therapy  Post therapy  p-value | 8.17±2.13  6.24±3.22  0.0737 | 2.27± 0.68  1.97± 0.62  0.2513 | 1.80±0.44  1.29±0.51  0.0216 | 5.45±1.38  3.86±1.16  0.082 | 7.98±1.83  6.58±1.56  0.0670 | 13.60±2.30  11.62±2.24  0.0550 | 14.94±2.33  12.97±2.93  0.071 | 5.23±1.78  3.92±1.57  0.061 | 7.79±2.07  3.86±1.79  0.0001 |
| Age- 30-50 (n=34)  Pre therapy  Post therapy  p-value | 8.00±2.59  3.96±2.27  0.0001 | 2.17± 0.50  1.68±0.42  0.0001 | 1.91±0.76  0.91±0.36  0.0001 | 5.91±2.91  2.90±0.76  0.0001 | 8.23±1.38  4.52±1.31  0.0001 | 14.59±2.67  8.32±2.11  0.0001 | 15.83±2.69  9.36±1.99  0.0001 | 5.62±1.28  3.36±0.74  0.0001 | 8.33±3.86  2.57±1.80  0.0001 |
| 51-70 (n=10)Pre therapy  Post therapy  p-value | 8.97±3.33  5.41±3.22  0.0182 | 2.45±0.53  1.54±0.44  0.0014 | 2.01±0.91  1.15±0.54  0.0163 | 5.81±3.21  3.40±1.21  0.0348 | 9.07±2.55  4.09±1.48  0.0001 | 13.18±3.01  8.39±1.23  0.0012 | 14.52±2.91  9.32±1.28  0.0006 | 4.95±1.03  3.32±.48  0.0004 | 7.09±2.10  2.38±1.01  0.0001 |
| Menopause Pre (n=28)  Pre therapy  Post therapy  p-value | 8.27±2.82  4.24± 0.42  0.0001 | 2.16±0.38  1.68±0.43  0.0001 | 1.93±0.79  0.93±0.39  0.0001 | 5.84±3.01  2.98± 0.81  0.0001 | 8.08±1.47  4.41±1.20  0.0000 | 14.31±2.75  8.38±2.31  0.0000 | 15.53±2.68  9.43±2.15  0.0000 | 5.51±1.31  3.32±0.70  0.0000 | 7.97±3.93  2.51±1.67  0.0000 |
| Post (n=16) Pre therapy  Post therapy  p-value | 8.13±2.75  4.38±2.84  0.0006 | 2.36±0.69  1.58± 0.43  0.0009 | 1.94±0.80  0.99±0.47  0.0006 | 5.97±2.94  3.08±1.05  0.0019 | 9.03±1.99  4.47±1.61  0.0001 | 14.20±2.93  8.25±1.09  0.0001 | 15.54±2.99  9.22±1.18  0.0001 | 5.40±1.17  3.41±0.68  0.0001 | 8.19±2.91  2.55±1.66  0.0001 |
| Tumor Size- T2 (n=09)  Pre therapy  Post therapy  p-value | 7.71±2.24  3.78±2.11  0.0003 | 2.32±0.44  1.48±0.29  0.0001 | 1.69±0.36  0.86±0.34  0.0001 | 4.61±0.65  2.76±0.86  0.0001 | 8.56±1.87  3.38±0.94  0.0001 | 13.36±2.41  6.41±0.95  0.0001 | 15.48±2.26  8.65±0.74  0.0001 | 5.14±0.94  3.11±0.48  0.0001 | 6.52±2.91  1.94±1.14  0.0003 |
| T3- (n=11) Pre therapy  Post therapy  p-value | 8.13±3.75  4.81±1.91  0.0209 | 2.18±0.42  1.55±0.51  0.0049 | 1.85±0.94  1.05±0.28  0.0140 | 5.49±3.19  3.14±0.98  0.0564 | 7.88±1.99  4.40±1.61  0.0001 | 13.43±3.01  8.82±1.68  0.0016 | 14.23±3.13  9.36±1.65  0.0008 | 5.39±1.48  3.38±0.67  0.0004 | 7.91±4.48  2.57±1.44  0.0016 |
| T4- (n=24) Pre therapy  Post therapy  p-value | 8.52±2.51  4.28±3.03  0.0001 | 2.21±0.60  1.77± 0.40  0.0065 | 2.09±0.84  0.95±0.51  0.0001 | 6.78±3.35  3.28±0.79  0.0001 | 8.63±1.51  5.41±1.41  0.0001 | 15.45±2.76  9.47±2.33  0.0001 | 16.26±2.69  9.69±2.24  0.0001 | 5.67±1.27  3.46±0.78  0.0001 | 8.94±3.26  2.79±1.92  0.0001 |
| Node- N0- (n=15)  Pre therapy  Post therapy  p-value | 7.82±1.78  3.51±2.21  0.0001 | 2.10±0.26  1.70±0.47  0.0039 | 1.77±.038  0.81±0.35  0.0001 | 5.22±1.27  2.63±0.69  0.0001 | 8.07±1.23  4.67±1.34  0.0001 | 14.66±1.93  8.21±1.65  0.0001 | 15.82±2.09  9.30±1.51  0.0001 | 5.66±1.17  3.42±0.60  0.0001 | 8.57±3.54  2.53±1.26  0.0001 |
| N1- (n=29) Pre therapy  Post therapy  p-value | 8.42±3.16  4.69±2.65  0.0001 | 2.30±0.60  1.61±0.41  0.0001 | 2.02±0.92  1.03±0.43  0.0001 | 6.24±3.48  3.21±0.93  0.0001 | 8.61±1.92  4.30±1.36  0.0001 | 14.07±3.15  8.41±2.11  0.0001 | 15.39±3.08  9.37±2.02  0.0001 | 5.37±1.30  3.32±.74  0.0001 | 7.78±3.60  2.52±1.83  0.0001 |
| Stage- IV- (n=44)  Pre therapy  Post therapy  p-value | 8.22±2.76  4.29±2.55  0.0001 | 2.23± 0.52  1.64± 0.43  0.0001 | 1.93±0.78  0.96± 0.41  0.0001 | 5.89±2.94  3.01±0.89  0.0001 | 8.42±1.72  4.43±1.35  0.0001 | 14.27±2.78  8.34±1.94  0.0001 | 15.53±2.76  9.35±1.84  0.0001 | 5.47±1.25  3.35±.69  0.0001 | 8.05±3.56  2.52±1.64  0.0001 |
| Hormone Status  ER+ (n=26) Pre therapy  Post therapy  p-value | 8.82±3.06  3.32±1.58  0.0001 | 2.25±0.51  1.57±0.29  0.0001 | 2.02±0.76  0.78±0.26  0.0001 | 6.15±2.89  2.06±0.58  0.0001 | 8.60±1.59  4.34±1.26  0.0001 | 14.62±2.71  7.90±1.14  0.0001 | 15.87±2.79  6.96±1.04  0.0001 | 5.61±1.48  3.30±.64  0.0001 | 8.53±4.19  2.17±1.34  0.0001 |
| ER- (n=18) Pre therapy  Post therapy  p-value | 7.34±2.04  5.69±3.04  0.0741 | 2.20± 0.53  1.75±0.56  0.0209 | 1.81±0.82  1.21±0.47  0.0081 | 5.52±3.07  4.15± 0.87  0.0816 | 8.16±1.90  4.56±1.49  0.0001 | 13.77±2.88  8.96±2.62  0.0001 | 15.05±2.72  11.91±2.54  0.0011 | 5.27±0.80  3.43±0.76  0.0001 | 7.36±2.33  2.93±1.93  0.0001 |
| PR+ (n=24) Pre therapy  Post therapy  p-value | 8.92±2.66  3.46±1.61  0.0001 | 2.20± 0.50  1.55±0.31  0.0001 | 2.02±0.70  0.81±0.27  0.0001 | 6.04±2.71  2.66±0.63  0.0001 | 8.26±1.17  4.19±1.27  0.0001 | 14.74±2.78  8.01±1.20  0.0001 | 15.92±2.87  9.05±1.13  0.0001 | 5.44±1.46  3.31±0.71  0.0001 | 8.02±4.13  2.51±1.57  0.0001 |
| PR- (n=20) Pre therapy  Post therapy  p-value | 7.51±2.74  5.12±3.05  0.0065 | 2.26±0.54  1.74±0.51  0.0036 | 1.84±0.87  1.11±0.48  0.0013 | 5.74±3.22  3.37±0.99  0.0025 | 8.58±2.14  4.66±1.40  0.0001 | 13.81±2.77  8.66±2.45  0.0001 | 15.15±2.66  9.65±2.34  0.0001 | 5.50±1.03  3.39±0.68  0.0001 | 8.08±2.98  2.54±1.75  0.0001 |
| HER2+ (n=25)Pre therapy  Post therapy  p-value | 8.38± 2.73  2.56± 1.57  0.0001 | 2.25±0.62  1.42± 0.32  0.0001 | 2.02±0.91  0.82±0.25  0.0001 | 6.27±3.61  1.50±0.63  0.0001 | 8.67±1.68  2.05±0.71  0.0001 | 14.22±2.26  7.67±1.12  0.0001 | 15.49±2.37  5.36±1.08  0.0001 | 5.22±1.20  3.18±0.74  0.0001 | 7.08±3.67  1.49±1.59  0.0001 |
| HER2- (n=19)Pre therapy  Post therapy  p-value | 8.02±2.86  6.18±3.27  0.0731 | 2.21±0.37  1.89±0.64  0.0673 | 1.82±0.60  1.12±0.51  0.0004 | 5.44±1.85  4.59± 1.13  0.0960 | 8.13±1.56  6.88±2.24  0.0535 | 14.34± 3.36  9.13±2.40  0.0001 | 15.58±2.13  13.47± 2.96  0.0609 | 5.77±1.27  3.56±0.58  0.0001 | 9.22±3.12  3.57±1.95  0.0001 |
