# Supplementary figures and images for: Circulatory Level of Inflammatory Cytoskeleton Signaling Regime Proteins in Cancer Invasion and Metastasis
Source: Front Oncol. 2022 Jul 7;12:851807. doi: 10.3389/fonc.2022.851807 (PMC9300851; doi:10.3389/fonc.2022.851807)

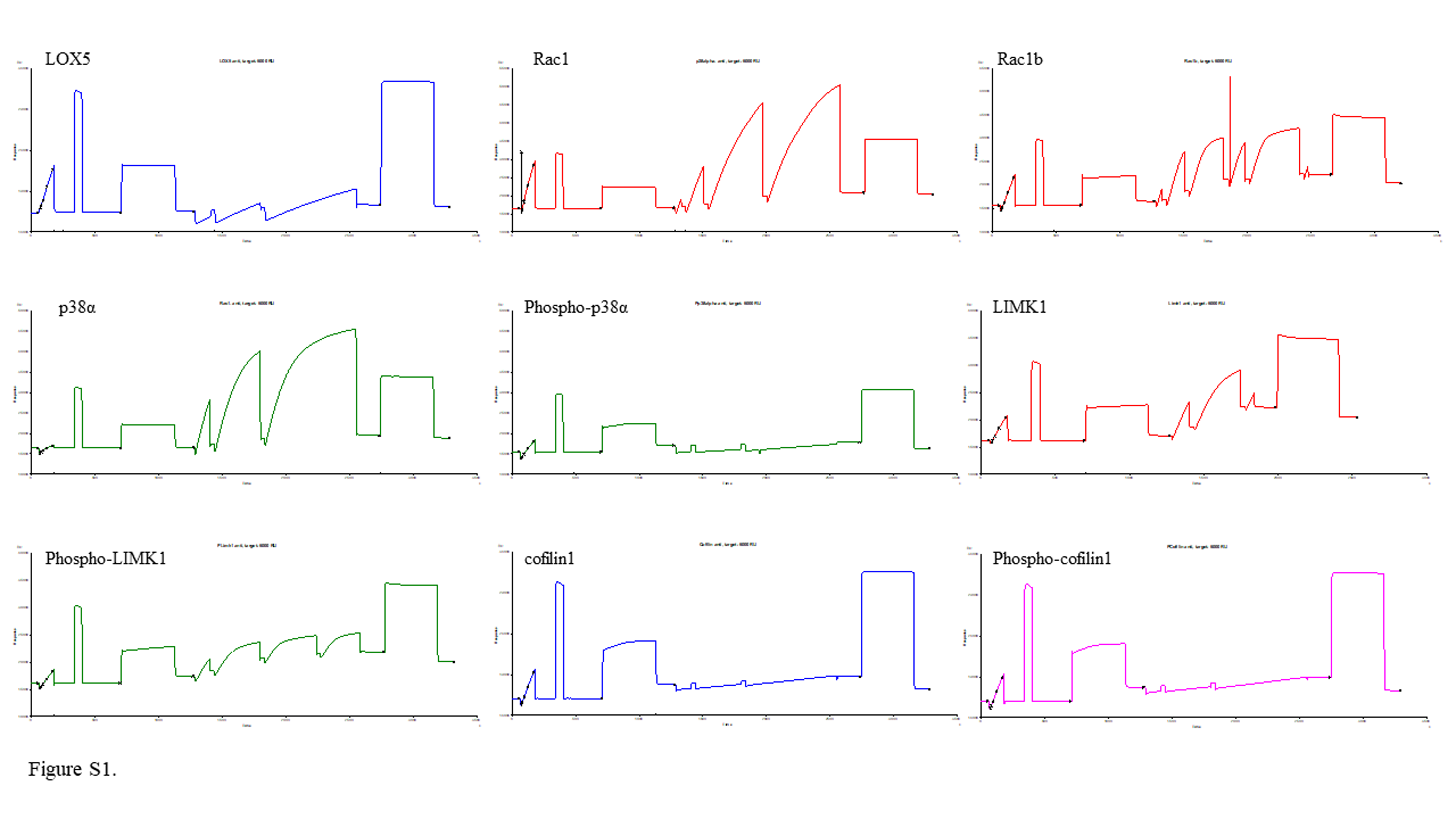

Supplement: Supplementary Figure 1 — Immobilization of LOX5, Rac1, Rac1b, p38α, hosphor-p38α (Y-182), LIMK1, hosphor-LIMK1 (T-508), cofilin1, and phospho-cofilin1 (S-3) antibodies on CM5 sensor chip. [file Image_1.tif]

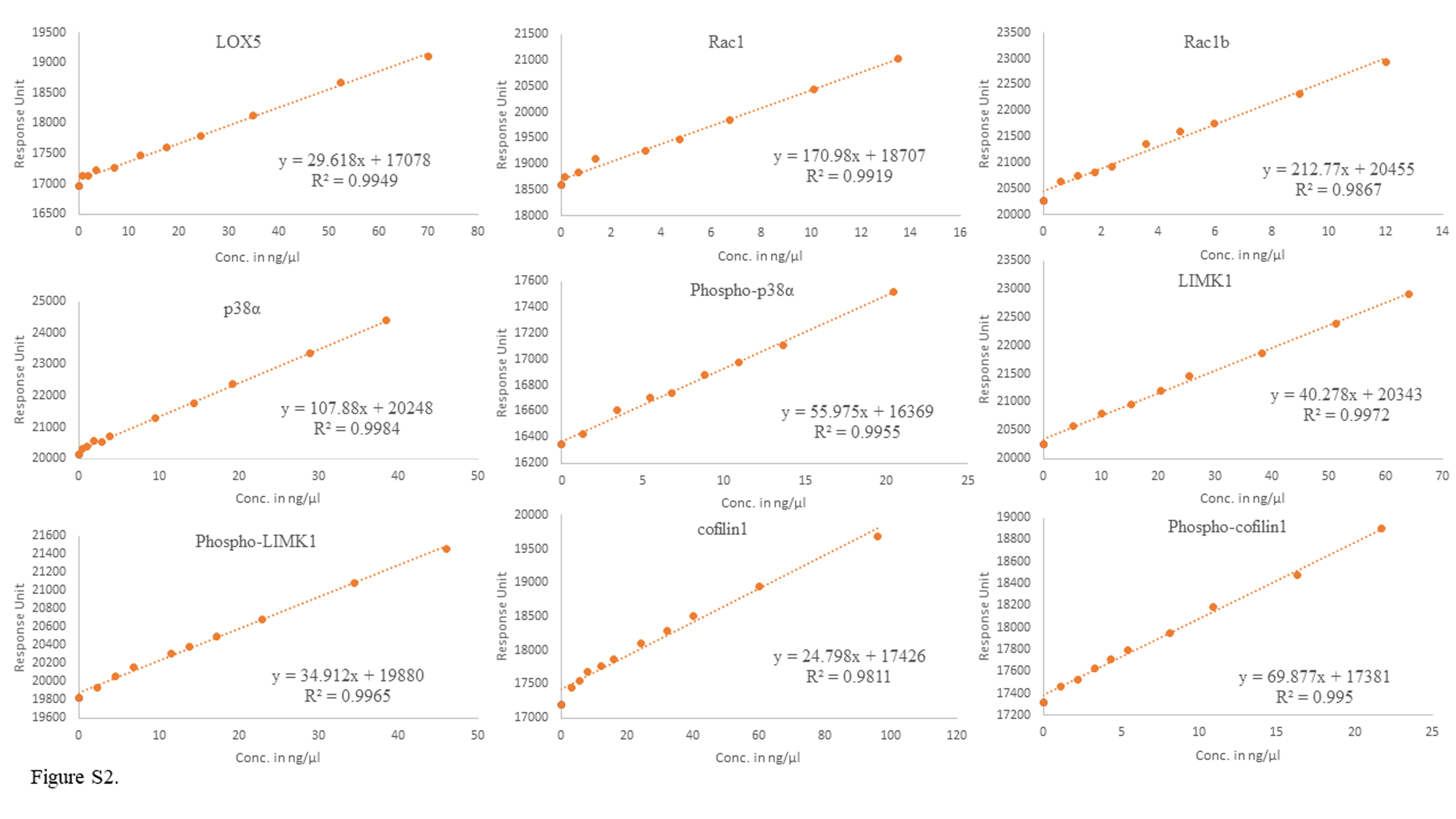

Supplement: Supplementary Figure 2 — Standard curve of LOX5, Rac1, Rac1b, p38α, phospho-p38α (Y-182), LIMK1, phospho-LIMK1 (T-508), cofilin1, and phospho-cofilin1 (S-3) proteins. [file Image_2.tif]
